# Improving the Thermal Performance and Energy Efficiency of Buildings by Incorporating Biomass Waste into Clay Bricks

**DOI:** 10.3390/ma16072893

**Published:** 2023-04-05

**Authors:** Sama Ahmed, Mohamed Esmat El Attar, Nasser Zouli, Ahmed Abutaleb, Ibrahim M. Maafa, M. M. Ahmed, Ayman Yousef, Ayman Ragab

**Affiliations:** 1 Department of Architecture Engineering, Faculty of Engineering, Aswan University, Aswan 81542, Egypt; 2 Department of Architecture Engineering, Faculty of Engineering, British University, Cairo 11837, Egypt; 3 Department of Chemical Engineering, Faculty of Engineering, Jazan University, Jazan 11451, Saudi Arabia; 4 Department of Mathematics and Physics Engineering, Faculty of Engineering at Mataria, Helwan University, Cairo 11718, Egypt

**Keywords:** bricks, clay, thermal insulation, energy efficiency buildings, biomass waste

## Abstract

Excessive urban construction is primarily driven by uncontrolled population growth, which has serious consequences for the environment, energy, cost, and human life in general when building materials are massively used. In terms of energy and economic efficiency, buildings that make use of sustainable construction materials and technologies perform better. This is because building in an eco-friendly way results in less waste. Agro-industrial by-products and insulating materials are two examples of sustainable materials that have been put to good use in the climate change mitigation effort and to preserve the environment. Precast components are emphasized as a viable option that is suitable for this purpose and may potentially fulfill the need for housing units. Thus, this study investigated the viability of employing agricultural waste consisting of pomegranate peel waste to produce fired clay bricks. Results demonstrated that the optimum amount of pomegranate peel waste was determined to be 15%, and the optimal firing temperature was determined to be 900 °C. The thermal conductivity of all test samples was lower than that of conventional brick. Furthermore, when compared to conventional wall brick, all the tested samples of manufactured brick reduced energy consumption by 17.55% to 33.13% and carbon dioxide emissions by 7.50% to 24.50%. In addition, the economic feasibility of employing each synthetic sample was evaluated by computing the simple payback time (SPP). It was determined that 1.88–10.74 years were required for the brick samples to provide a return on their initial investment. Due to its ability to decrease heat gain, preserve energy, minimize CO_2_ emissions, and shorten the payback time, burned clay bricks manufactured from pomegranate peel waste are regarded as a feasible building material. Hence, manufactured bricks are usually considered an exceptional contribution to environmental sustainability.

## 1. Introduction

The increasing worldwide population necessitates a rise in energy production to meet the demands of contemporary life. Since 1950, there has been a dramatic increase in the world’s population, and with it, a corresponding increase in demand for energy. Most of this growth has happened in developing countries, which already have to deal with acute energy shortages.

There were 7.7 billion people on Earth in 2019, and experts predicted the number would rise to 8.5 billion by 2030. It is projected that between 2019 and 2030, the resident populations of Sub-Saharan Africa (which now has a very small population) and Central and Southern Asia (which also has a relatively small population) would grow by 0.3 billion and 0.2 billion, respectively. There will likely be 9.7 billion people on Earth by 2050, according to statistics [1]. Energy is becoming more important on a global scale. The developed world continues to use vast amounts of energy, despite the growing need in developing countries. The rate of global population growth remains constant. The growing human population and improved living conditions in many developing nations will drive up the need for energy. The highest energy use is seen in the most populous and industrialized countries. This is because of the region’s dependence on the industry, its broad use of cars, and its reliance on home consumption owing to families having multiple home devices [1].

While the majority of the globe shifts to low-carbon energy sources, it falls on developing countries to provide for the billions of people who still lack access to the most basic forms of modern energy [2,3].

As the world’s population and per capita consumption continue to rise, it is urgent to create sustainable patterns of consumption and production that meet the basic and immediate needs of a growing population [4]. Contemporary studies in the fields of recycling and energy conservation are primarily motivated by the desire to lower cooling costs in hot, arid regions by finding new uses for industrial and agricultural wastes to produce inexpensive construction materials that restrict the flow of heat inside buildings. Numerous studies have examined the potential of industrial and agricultural wastes as raw materials for a wide range of building products, and many have found promising applications [5]. Some examples of such materials include sludge, polystyrene, fly ash, mushroom compost, sugar cane husk, and polystyrene. One practical solution to the energy crisis is insulation built from recycled building materials.

Since exterior wall insulation has been neglected in favor of isolating ceilings alone, there has been an increased demand for clay bricks with enhanced insulating performance. One way to enhance the insulating qualities of brick is to introduce porosity into the clay body. Sawdust, polystyrene, paper sludge, coal, and agricultural wastes are all examples of organic materials often used as pore formers [5,6,7]. The use of organic product wastes as a pore-forming agent in the brick industry decreases the clay’s density and, in general, increases the clay’s thermal insulation potential.

Numerous plant-based waste products, such as coal [8], olive pomace [9], mushroom fertilizer waste [10], sugar cane husks, rice husks [11], and sawdust, have been investigated as potential additions to the brick production. Additionally, chemicals used to improve the structural strength of bricks are discussed in a wide variety of synthetic articles [12,13,14,15]. When organic matter is added to bricks, the bricks become more porous and better able to retain heat. There have been some interesting experiments, but because they are often one-offs, they have not been used to make bricks on a large scale.

The physicochemical and acoustic characteristics of powdered potato peels were studied by Ghorbani et al. [16]. The addition of potato peel powder (PPP) to the bricks increases their porosity, which in turn provides better sound insulation by reducing the intensity of vibrations. While this was happening, the addition of sour orange leaf SOL powder to the clay lowered the rate at which heat was transferred. This, when applied at a concentration of 7%, has the potential to decrease thermal conductivity by 76.2%.

Eliche et al. [9] replaced 10–50% of the clay in brick manufacturing with olive pomace bottom ash in order to examine brick qualities and provide an innovative technique to recycle olive pomace bottom ash. Bottom ash from olive pomace (up to 20% weight) has been shown to be a viable clay substitute to produce high-quality bricks, while also reducing pollution and conserving natural resources.

By combining various amounts of sugarcane bagasse ash (SBA) and rice husk ash (RHA) per weight of clay, Syed Minhaj et al. [11] produced thermally efficient clay bricks from agricultural waste. This study demonstrates that by incorporating SBA and RHA (up to 15% by clay weight) into the manufacturing process of burned clay bricks, they could reduce the amount of waste sent to landfills while simultaneously creating environmentally friendly, energy-efficient bricks. Amin Al-Fakih et al. [12] recently reported the most up-to-date study results on recycling brick manufacturing waste. Recycling waste into bricks for use in masonry could help create more sustainable and long-lasting building materials.

Cecile Bories et al. [13] looked at the impact of different pore-forming agents produced from renewable or mineral resources on the physical, mechanical, and thermal characteristics of clay bricks. It’s possible that waste sent to landfills and the use of hazardous materials could be reduced if recycled materials were used in building projects.

Bricks built using wood ash or rice husk ash (10–30% by weight) instead of clay were compared to bricks prepared with clay alone by Eliche et al. [17]. The findings demonstrate that bricks containing 20% wood ash may be made that are technically feasible, have mechanical qualities that are on par with control bricks, and have a 15% decrease in heat conductivity relative to untreated bricks. Adding rice husk ash and wood ash to the clay bricks made them significantly lighter.

Sawdust, tobacco ash, and grass were used by Ismail Demir [18] to investigate the effectiveness of incorporating different amounts of organic wastes into clay bricks (0%, 2.5%, 5%, and 10% in wt.). The results of the experiments on form, plasticity, density, and mechanical properties were studied. He found that bricks may be produced using organic pore-forming materials such as sawdust, tobacco waste, or grass with minimal environmental effect.

Milica et al. [19] investigated the use of organic and inorganic industrial wastes in clay bricks to see if it would be beneficial and distinguish them from standard clay substitutes. Bricks produced using the analyzed wastes as secondary source materials were found to have better insulating properties. Organic additives had a significant impact on the quality of burned items, whereas inorganic additives had a minor impact.

In terms of total production, Egypt ranks seventh globally in pomegranate production [20]. Egypt ranks fifth among the world’s top exporters [21] due to its large harvests and high export rates of medicines and fruit juices made from this crop, which is grown on an area of roughly 31,987 hectares and generates a total of 382,587 tonnes. However, pomegranate waste products such as peels and rinds are not recycled or composted.

Based on the aforementioned observations, pomegranate peel waste (PPW) could be used to manufacture fired clay bricks with improved porosity and thermal performance. So, the goal of this study is to figure out the best amount of pomegranate peel waste and firing temperature for a fired clay brick that has better thermal conductivity and uses less energy.

## 2. Materials and Methods

**Figure 1:**
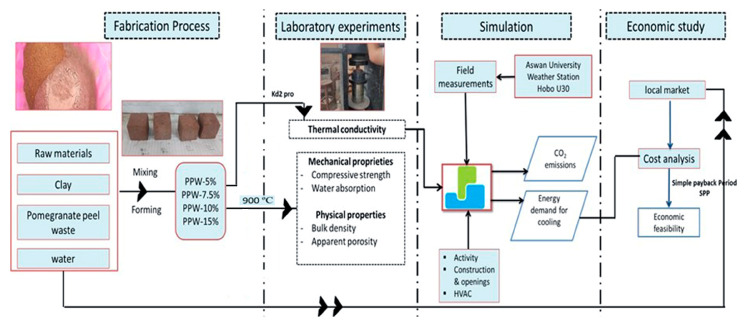
The study framework.

The study investigated the efficacy of clay bricks with pomegranate peel waste (PPW) incorporated into the external walls in the present climate of the new city of Aswan. Specifically, there were four fundamental stages to the study. As can be seen in Table 1, the first phase included the creation of brick samples with differing percentages of pomegranate peel waste at a temperature of 900 °C. Following this, the second step involves conducting the testing of brick samples for their mechanical, physical, and thermal characteristics. In the third step, Design Builder simulation software was used to determine what further energy was saved by using the different brick samples. In the last stage, the financial viability of construction using pomegranate peel trash was examined. A flowchart of the methods used in this investigation is shown in Figure 1.

### 2.1. Study Area

**Figure 2:**
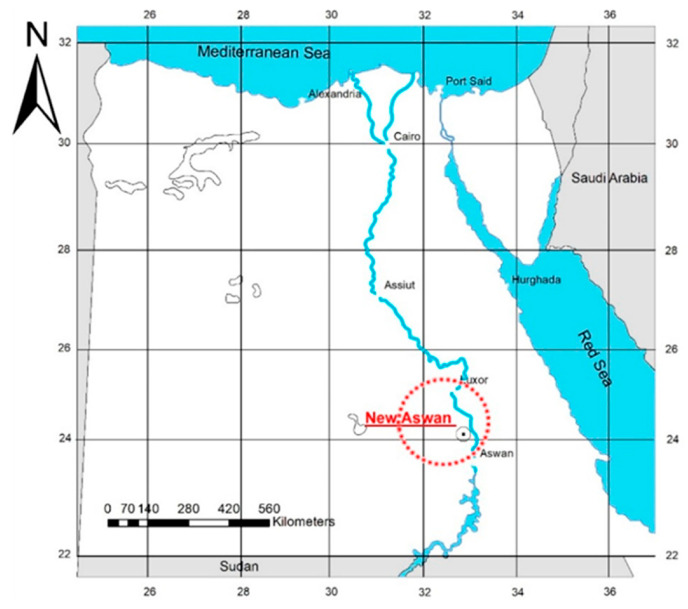
Location of New Aswan city [26].

This study focused on a hot, dry climate because of the region’s increasing energy demands for cooling and the limits of current climate solutions. Homeowners and others who build their own houses sometimes neglect insulation in Egypt [22,23,24]. Government-built buildings often have their roofs but not their walls insulated. The study set out to determine whether using clay bricks composed of pomegranate waste would improve thermal performance and reduce energy consumption in the proposed new city of Aswan. Located in the western Nile Valley, about 10 km from the present-day city of Aswan, New Aswan is easily accessible (as depicted in Figure 2). It has the characteristic hot desert climate of Egypt, with summer highs exceeding 45 °C and winter lows below 15 °C. May and June have the lowest relative humidity (12%), while December and January have the highest (36% and 34%, respectively) [25].

### 2.2. Raw Materials and Fabrication Process

#### 2.2.1. Materials

**Figure 3:**
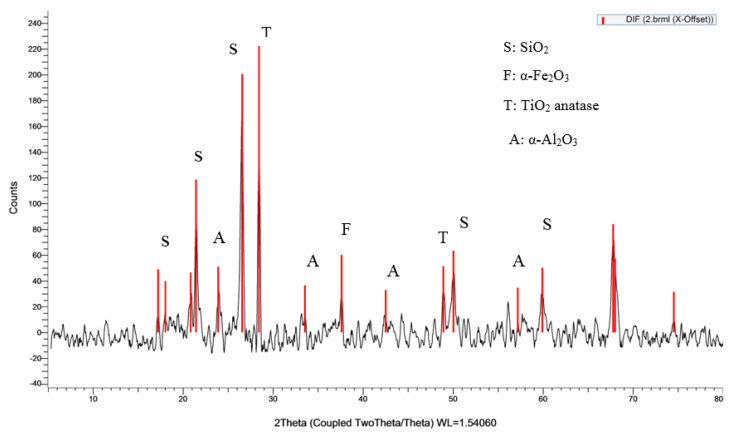
XRD of clay.

**Figure 4:**
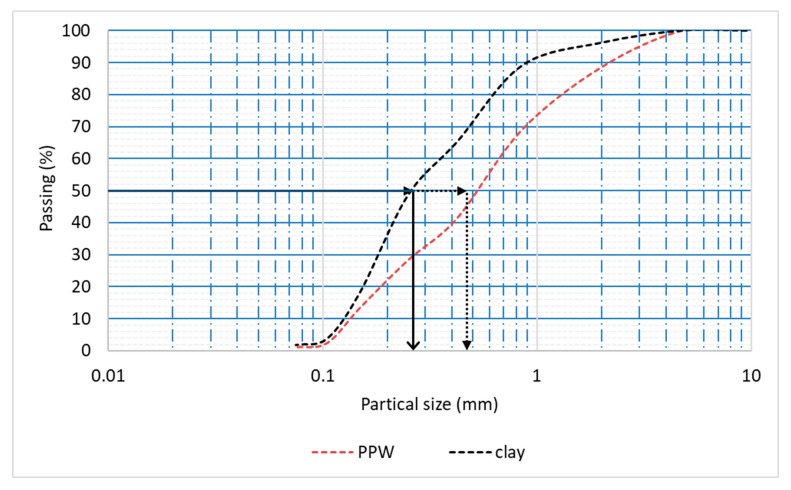
Sieve analysis of clay and PPW.

Clay was used as the primary brick component in this study. Table 2 displays the results of X-ray fluorescence (XRF) analysis on the clay. As can be seen in Figure 3, the dominant mineral phase of the clay was determined with the use of X-ray diffraction (XRD). Analysis by X-ray diffraction indicates that kaolin predominates among the minerals in the combination. PPW is collected from local juice shops. It is dried under normal sunlight. It is then milled to obtain fine particle powder. The powder was washed with water to remove any undesirable dust and dried in an oven at 100 °C for two days to obtain fully dried powder before being mixed with clay. The XRF of PPW is listed in Table 2. Because of its high organic content, PPW has a greater LOI. The particle size analysis of clay and PPW is shown in Figure 4; the average diameter of the clay particles was 0.27 mm, while the PPW particles were 0.47 mm in diameter.

#### 2.2.2. Samples Preparation

**Figure 5:**
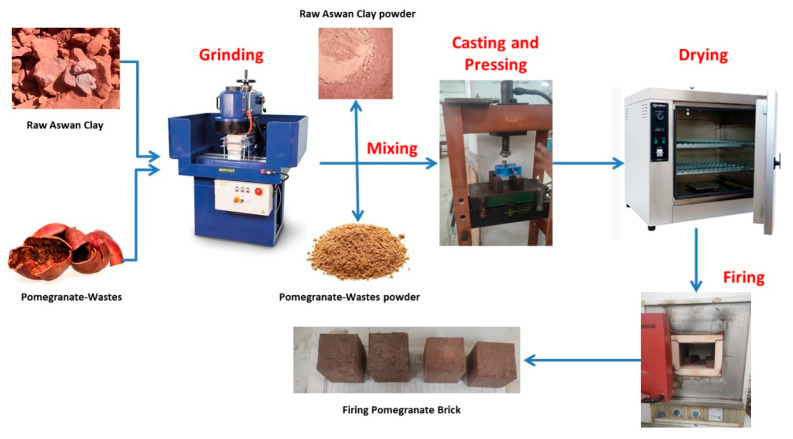
Samples preparation steps.

A ball mill was used to finely grind the derided PPW and mix it with clay at different percentages (0%, 5%, 7.5%, 10%, and 15%). The different mixed formulations were blended together for two minutes to obtain a consistent dry mixture. The added water to prepare the paste is 20 wt.% based on the mixtures. The formed pastes were pressed into 50 mm cubic molds. After filling the molds with the mixture, they were compressed at three intervals to remove any remaining air bubbles. After 24 h of air drying, the samples were removed and dried at 120 °C for 6 h. The dried samples are fired at 900 °C for 4 h at 10 °C/min heating rate. The low heating rate is to avoid the rapid escape of CO_2_ and H_2_O during the firing process. The brick preparation procedure was presented in Figure 5.

### 2.3. Laboratory Experiments

This section is divided into subsections that focus on several tests such as compressive strength, water absorption, density, apparent porosity, and thermal conductivity.

#### 2.3.1. Compressive Strength

For the purpose of this calculation, the compressive strength of the specimen was determined as per ISO 9652 [27].

#### 2.3.2. Water Absorption, Density, and Apparent Porosity

ISO 5017 [28] was used to assess absorption. After being burned, the bricks spent 5 h in a hot water bath before being left to cool to room temperature. After being removed from the water, the specimens were dried carefully using a clean cloth. The water absorption was calculated according to equation in the literature [29]. Density is a major determinant in how effectively burned clay brick’s function. The low density of the burnt bricks contributes to their poor thermal conductivities and the low dead loads of the building. Bulk density may be calculated according to literature [30]. There is a strong correlation between the perceived porosity of burnt clay bricks and their ability to absorb water. Fired clay bricks with a high porosity are great insulators because of their poor thermal conductivity. Apparent porosity may be calculated according to literature [31], which describes the percentage connection between the volumes of open pores and the external volume.

#### 2.3.3. Thermal Conductivity

Thermal conductivity was measured using the KD2 Pro Thermal Properties Tester on brick samples in line with ASTM D 5334 [32]. By use of a transient heat conduction technique, the tester digitally records the thermal conductivity at room temperature.

### 2.4. The Simulation Procedures

Each day was planned by collecting the daily activities that reflect the traditional Egyptian style of living (holidays, work hours, etc.). Recent demographic statistics [33] indicate that the typical Egyptian family has five members. In addition, the residents of the building will most likely range in age from 22 to 60 years. The building schedule was designed according to the vacations. Egypt celebrates a number of festivals. Egyptians work from 8:00 a.m. to 3:00 p.m. every day of the week. During the week, the great majority of the building’s owners will be at their places of employment. As a result, the simulation software was provided by the typical family’s daily activities, including household tasks. According to a prior study [34], the average power intensity of lighting in the living room is 17 W/m^2^, whereas the average power intensity in the bedroom is 13 W/m^2^. The average power intensity for the lighting at these locations is 9 W/m^2^. The Design-Builder software was supplied with information on the heat gain from the appliances placed in each flat. Furthermore, all needed input data for the simulation software was presented in Table 3.

#### 2.4.1. The Model Definition

**Figure 6:**
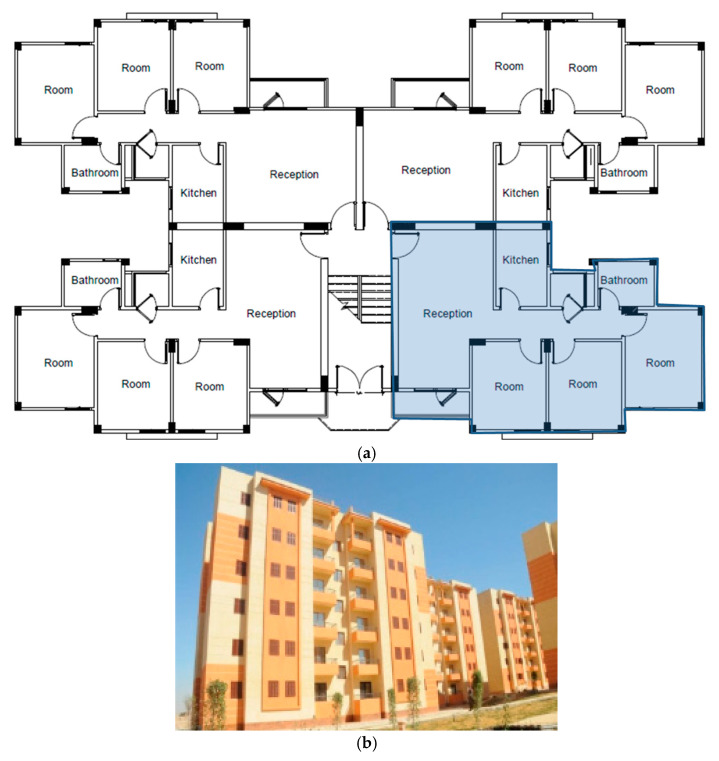
The Study model: (**a**) Ground floor plan; (**b**) the building facade.

The building was established as a replica of a residence located in New Aswan City. Due to its location within the public housing complex, this building was chosen despite its lack of consideration for local climate and its use of insulation methods other than roof insulation. Each apartment is around 86 m^2^ in area, and the entire building, including the stairwell, is about 357 m^2^ in the area as shown in Figure 6. There are six stories and four apartments on each floor. There was 3 m between the floor and ceiling, and the ratio of windows to walls was roughly 10% of the total façade area.

#### 2.4.2. Weather Data File

**Figure 7:**
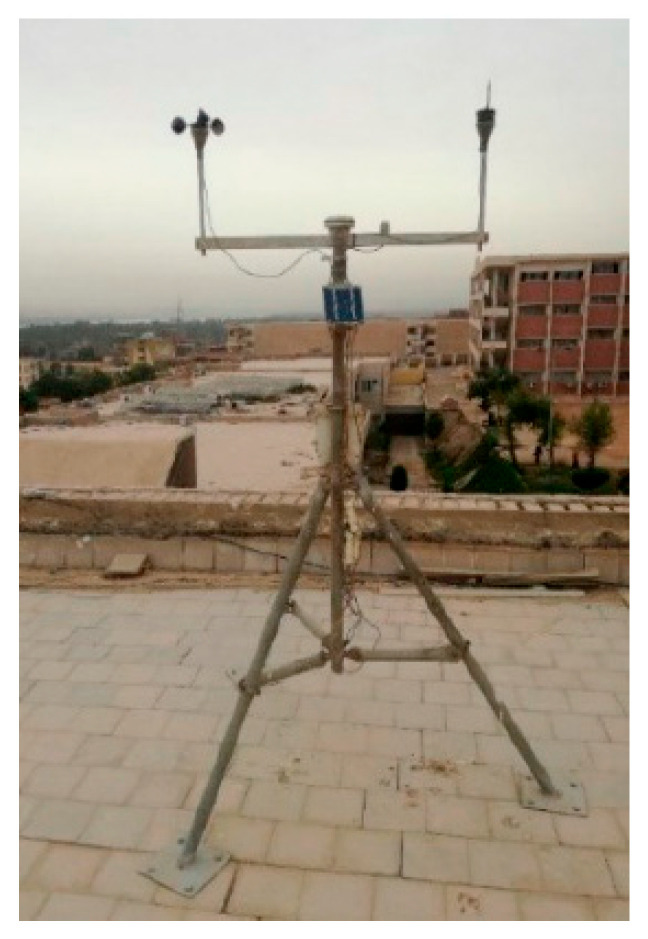
The weather station at Aswan University (Hobo U30).

The modeling software reportedly comes with the 2002 epw file (Energy Plus Weather) for the Aswan climate zone, as stated on the website of the United States Department of Energy (DOE). These epw files are textual CSV file that provides hourly weather data at the location of the research for a whole year’s worth of the calendar year. the standard weather data file in the software was replaced with the in situ weather data file for Aswan climatic conditions (2021), which was obtained from the Aswan University weather station (Hobo U30) as shown in Figure 7. This was performed with the purpose of simulating the conditions that would be encountered in the real world. It was necessary to first convert the original epw file that has the weather data to a CSV file before getting various extracted meteorological data from the weather station. This was necessary in order to receive real data.

The dry-bulb temperature, relative humidity, global radiation, wind speed, and wind direction were all extracted as input variables from the weather station. In order to ascertain the dew point and the direct radiation, the element software was put to use. After that, the study utilized the newly created epw to import the revised CSV into the Design Builder application.

#### 2.4.3. Model Validation

**Figure 8:**
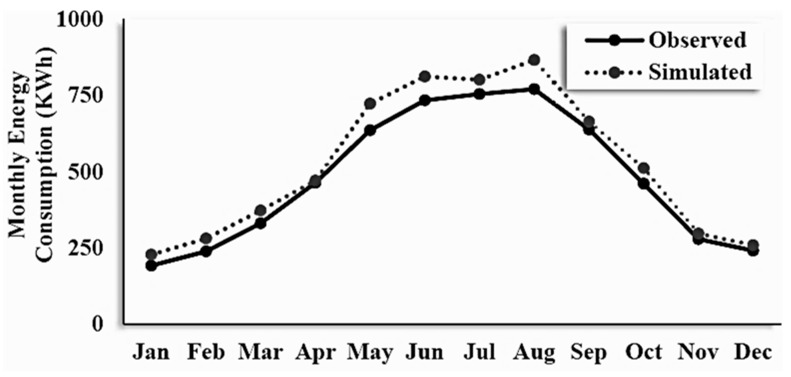
The validation of simulation results.

In accordance with ASHRAE Guideline 14-2002, the findings of the simulation were validated by modeling the required parameters of the research site. These factors included comfort zone limitations, illumination, building materials, and heating, ventilation, and air conditioning (HVAC) configurations. In order to accurately depict the overall energy consumption of all of the apartments in the building, the electricity bills from the apartment on the third level were used to make a comparison between the findings of the simulation and the actual energy usage. It was found that the results of energy simulations had an inaccuracy of 10.19%, as shown in Figure 8, and there was a correlation coefficient of 0.995 between the simulated values and the real values that were observed.

### 2.5. Electric Energy Prices

At a rate that was established by the Egyptian Ministry of Electricity and Renewable Energy for the residential sector, the cost of energy consumption per flat per month was calculated in Egyptian Pounds (EGP). Noting that electricity prices are increasing, the increases are applied in July of each year. The cost of energy was divided into seven categories. Where the first category of energy usage ranges from 0 to 50 kWh and costs around 0.58 Egyptian pounds. The second category consists of 51 to 100 kWh and costs around 0.68 EGP per unit. The third category includes energy usage ranging from 101 to 200 kWh at a cost of 0.83 EGP per kwh. The fourth category is between 201 and 350 kWh and costs 1.11 EGP per unit of energy. The fifth category is between 351 and 650 kWh at a cost of 1.31 per kWh. The sixth category ranges from 651 to 1000 kWh and costs around 1.36 EGP per kWh. Where the seventh category includes energy use that exceeds 1000 kWh and costs around 1.45 EGP.

## 3. Results and Discussion

The findings of this study are divided into five main major sections. The Section 3.1 includes the extracted mechanical characteristics of the manufactured brick, as well as a comparison between these extracted values and the Egyptian code to determine the percentages of compliance. The Section 3.2 investigates the physical attributes of the brick samples that were manufactured. The Section 3.3 contains the data for the thermal conductivity of manufactured brick. The Section 3.4 displays the findings of the simulation process in terms of the analyzed building’s internal thermal performance, cooling energy consumption, and related CO_2_ emissions. In the Section 3.5, the study was expanded to assess the economic viability of employing bricks containing pomegranate peel wastes to achieve optimal interior temperature conditions while reducing the initial cost by a significant amount.

### 3.1. Mechanical Properties

#### 3.1.1. Compressive Strength

**Figure 9:**
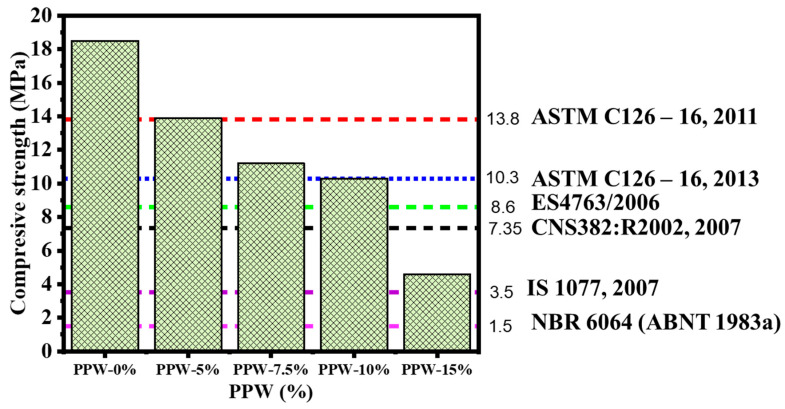
Effect of PPW addition on the compressive Strength of fired bricks at 900 °C.

Fabricated lightweight-fired bricks were tested for their compressive strength, and the findings are shown in Figure 9. Adding PPW lowered the compressive strength of the burnt clay at 900 °C from 18.5 MPa to 13.9, 11.2, 10.3, and 4.6 MPa when PPW-5%, PPW-7.5%, PPW-10%, and PPW-15% were added, respectively. This could be due to the formation of porous resulted from the PPW combustion. The samples that have been fired at 800 °C from 700 °C showed lower compressive strength compared to the samples fired at 900 °C. There has been a lot of research showing that as combustion temperatures rise, the density of the brick structure improves, and hence the compressive strength of fried bricks [35]. Furthermore, increase in the temperature leads to formation of glass phase from SiO_2_, which leads to eliminate the formed pores. The compressive strength of brick specimens for engineering applications ranges between 5 and 8 MPa [36,37,38]. According to various international building standards. All of the fabricated bricks in this investigation had greater compressive strength and were in accordance with international building standards. All fabricated bricks agree with the Brazilian Standard NBR 6064 (ABNT 1983a) [39], Indian Standard (IS 1077, 2007) [40], Chinese National Standard (CNS382:R2002, 2007) [41], and the Egyptian Standard (ES4763/2006) [42], while the samples composed of PPW-0%, PPW-5%, and PPW-7.5% agreement only with ASTM C62–13a, 2013 [43].

#### 3.1.2. Water Absorption

**Figure 10:**
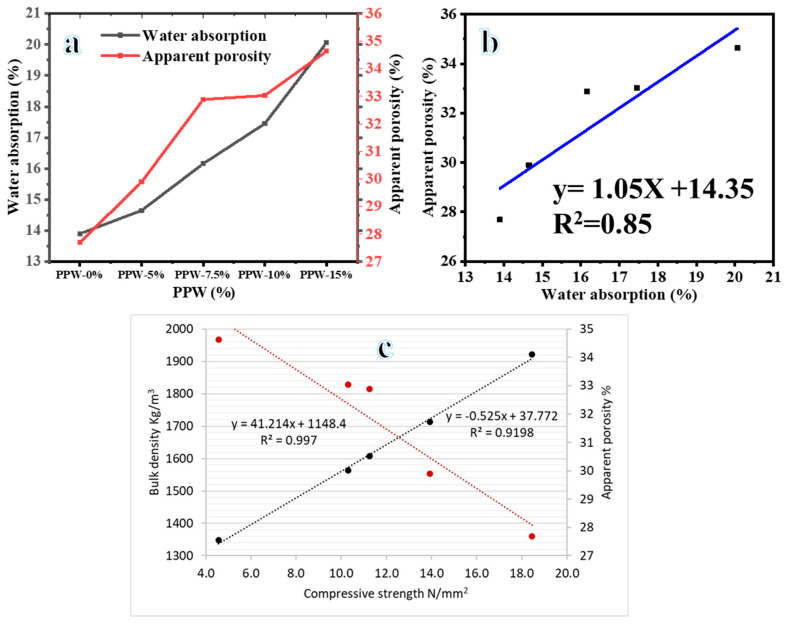
Effect of PPW addition on water absorption and apparent porosity (**a**), relationship between Water absorption and apparent porosity (**b**), and relationship between compressive strength, density, and porosity (**c**) of fired bricks at 900 °C with PPW waste additive.

Water absorption and porosity of lightweight brick fired at 900 °C and at rates of 0–15% clay substitution ratios are shown in Figure 10a. They are considered an indicator of a brick’s durability; this may also be used as a proxy for the surface’s actual material composition. They rise almost linearly with increasing PW content, demonstrating PW’s efficacy as a pore-forming agent. The absorption was increased by 22.6%, 21.1%, and 20.0% when PPW-5%, PPW-7.5%, PPW-10%, and PPW-15%, respectively, replaced the clay, compared to 13.5% at PW-0%. As known, the porosity is directly proportional to the water percentage Figure 10b. They considered the most important factor in the physical and mechanical characteristics of bricks. The greatest insulation could be achieved at suitable high porosity of clay bricks. The maximum water absorption is 20% at PPW-15%. This is in agreement with Moderate and normal weathering clay building bricks ASTMC62, in which the maximum water absorption is 22% and no limit, respectively [44]. The compressive strength, porosity, and bulk density of brick samples are linearly related to one another as shown in Figure 10c. If you know the porosity and density of a brick, you may use this correlation to estimate its compressive strength.

### 3.2. Physical Properties

#### Bulk Density

**Figure 11:**
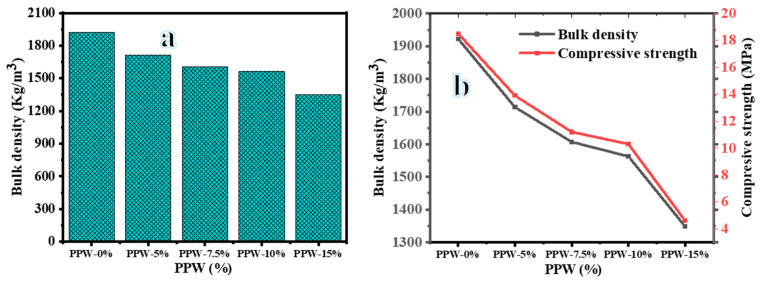
Effect of PPW addition on the bulk density (**a**) and the relationship between PPW, bulk density, and compressive strength (**b**) of fired bricks at 900 °C.

In the same manner of compressive strength, the brick samples containing PPW have a lower density than PPW-0% brick samples. As shown in Figure 11a, as the PPW percentage increased the bulk density decreased. This is due to eliminating the organic materials and mineral hydrates or carbonates in PPW that are decomposed at the suggested fired temperature, producing in an increase in pore size, and hence a reduction in density. When the fire temperatures were decreased from 900 °C to 700 °C, there was little change in the bulk density of the lightweight bricks was observed. The increase in the fire temperature leads to an increase in the bulk density. If the brick’s bulk density is less than 1680 kg/m^3^, as specified by the ASTM C90 standard [45], then it is called lightweight. Figure 11b shows the decreased bulk density and compressive strength with an increase in the PPW percentage.

### 3.3. Thermal Conductivity

**Figure 12:**
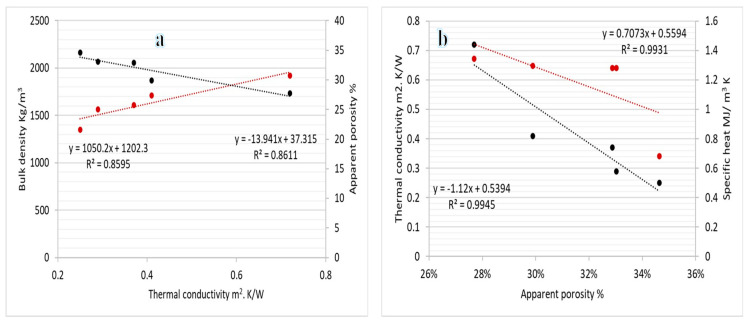
Relationship between thermal conductivity, density, and porosity (**a**) and relationship between porosity, thermal conductivity, and density (**b**) of fired bricks at 900 °C with PPW waste additive.

The study outcomes indicate that the best results were obtained when the manufactured bricks were fired at 900 °C, so the study investigated the thermal conductivity of all samples that have been fired at this temperature. (Conventional brick, PPW-5%, PPW-7.5%, PPW-10% and PPW-15%), and the results were as shown in Table 4, where the thermal conductivity decreases with increasing the proportion of pomegranate peel waste in the sample. The lower thermal conductivity of bricks may be due to the increased porosity and decreased density of the specimens with PPW. There is a linear relationship between thermal conductivity, porosity, and density of manufactured brick specimens, as shown in Figure 12a. The thermal performance of bricks with PPW can be predicted using this relationship. All the samples have thermal conductivities that are lower than those of conventional bricks. Thus, PPW bricks can be utilized in masonry structures, which will lead to the construction of green, low-energy buildings. Poor energy transfer and increased heat resistance are the direct consequence of the brick’s matrix developing larger pores as a result of increasing amounts of additives being added in. The specimen’s thermal resistance and thermal insulating characteristics, as measured by the reduction in thermal conductivity and the increase in specific heat capacity, improve with increasing porosity as PPW additive amounts are raised. This explanation is consistent with experimental results showing a rather high correlation coefficient between porosity level and the sample’s insulating capabilities as depicted in Figure 12b.

### 3.4. Simulation Results

#### 3.4.1. Thermal Performance of the Brick Contains Pomegranate Peels Waste

**Figure 13:**
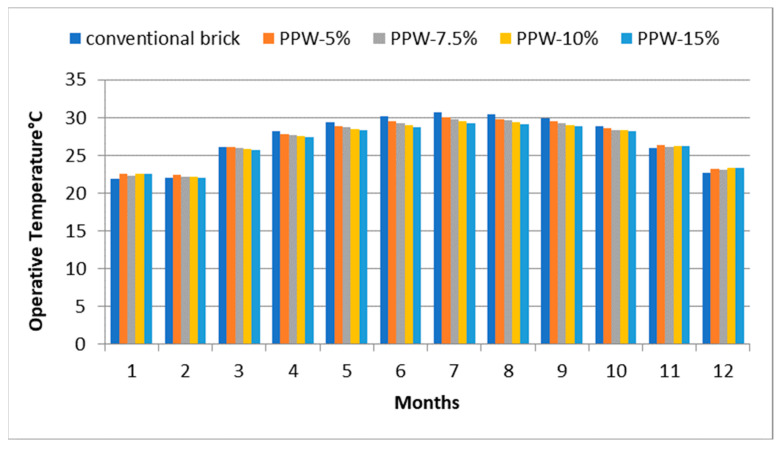
The operative temperature by using different proportions of pomegranate waste.

The thermal performance of PPW samples was evaluated using Design-Builder and compared to the thermal performance of conventional brick by calculating the operative temperature, which indicated an interior thermal comfort index that reflected the blending of air temperature influences. As depicted in Figure 13, the findings indicate that the operative temperatures of the brick samples varied. Throughout most of the year, the operative temperature drops since the percentage of pomegranate peel waste in the bricks increases. However, during the winter months, the temperature rises, perhaps due to a reduction in the thermal conductivity of the bricks. In July, the hottest month of the year in Aswan, the findings indicated that the temperature difference between PPW-15% bricks and conventional bricks was 1.47 K. During the same month, samples PPW-5%, PPW-7.5%, and PPW-10% recorded 0.71, 0.98, and 1.26 K, respectively. Throughout the rest of the year, the temperature decrease varied between 0.9 and 1 K.

#### 3.4.2. Evaluation of Annual Energy Demand for Cooling

**Figure 14:**
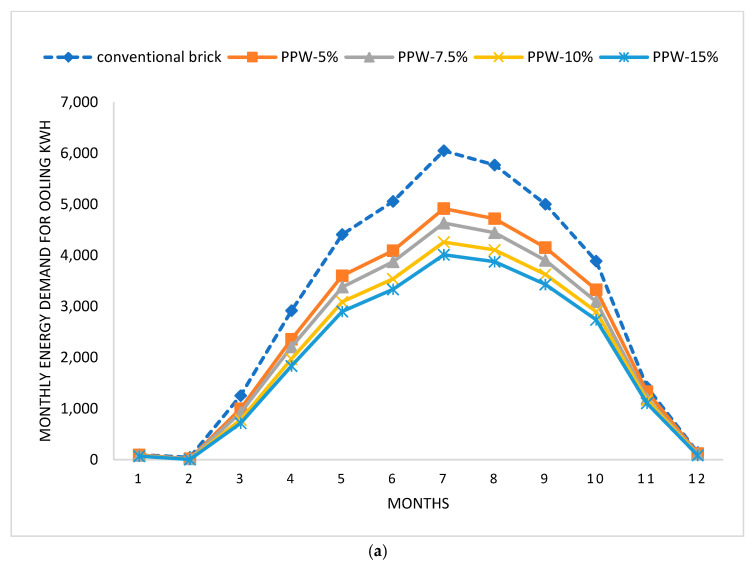

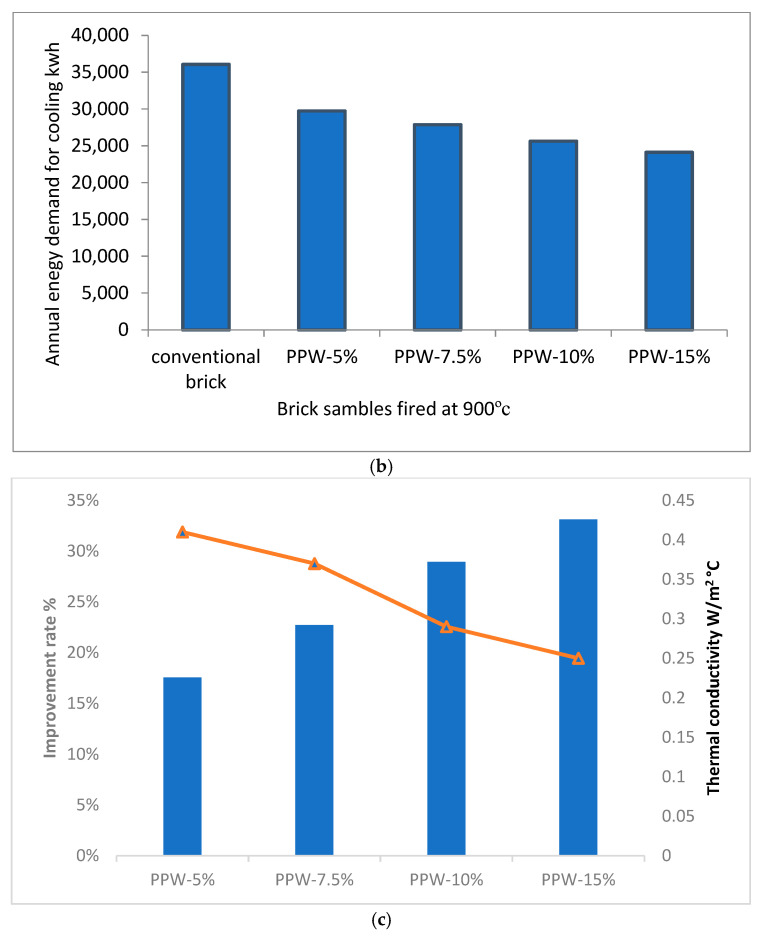
The cooling energy needed of each proposed brick sample, as predicted by simulation. (**a**) the monthly energy needed for cooling, (**b**) the annual energy demand for cooling. (**c**) Percentage of decrease in energy consumption by using different proportions of pomegranate peel waste.

The Design-Builder thermal simulation software was used to assess the impact of bricks containing varying percentages of pomegranate peel waste on the cooling loads of a residential building. The findings of the simulation, which are shown in Figure 14, showed how various formulations of pomegranate peel bricks influenced cooling energy needs.

Figure 14a shows that the summer months of April through October had the highest monthly variation in energy use across all assessed brick samples. In addition, the conventional brick represents the worst-case situation in terms of cooling energy usage. Each brick manufactured from pomegranate peel waste requires substantially less cooling energy than conventional bricks. July, the hottest month of the year, is the month in which cooling energy consumption reaches its highest level. During this time of year, Aswan may reach temperatures over 45 °C. Figure 14b,c show that when conventional bricks are replaced with a PPW-15% brick sample, the building’s cooling needs decrease by 11,946.1 kWh, representing an improvement rate of about 33.13% compared to the base scenario (conventional brick). PPW-10% decreased the amount of energy used for cooling by 10,437.2 kWh, or an improvement of 28.95%. PPW-7.5% and PPW-5% have the ability to reduce cooling energy consumption by 8192.44 kwh and 6327.34 kwh, respectively, with corresponding improvement rates of 22.72% and 17.50%. Considering that space cooling contributes 37–42% of a building’s total energy requirement [46,47], this will greatly lower the building’s energy consumption.

#### 3.4.3. The Effect of Proposed Fabricated Clay Brick Incorporating Pomegranate Peel Waste on the CO_2_ Emissions

In recent years, there has been an increase in the amount of embodied carbon dioxide that is part of the total life-cycle impact of buildings. This is the case even though technological advancements have resulted in a reduction in the amount of carbon dioxide that is produced during operation. Heating, air conditioning, ventilation, and lighting are the primary contributors to operational carbon dioxide emissions, while material extraction, fabrication, transportation, construction, maintenance, and demolition are the primary contributors to embodied carbon dioxide emissions [48]. According to the findings of this research, there is a relationship between the operational production of carbon dioxide (CO_2_) and the increasing usage of air conditioning in buildings located in hot and dry locations. There is a decent possibility that materials used in construction are adding to the ever-increasing quantities of embodied and operational carbon dioxide in the atmosphere. However, a lot of research has been done to improve building materials like concrete blocks, roof tiles, and wall bricks. This has led to better thermal performance, less energy needed to cool the building, and fewer CO_2_ emissions from the building’s operations.

The findings of this study show that the use of PPW bricks has the potential to improve the health of the people who occupy buildings by reducing the need for air conditioning and the rate at which heat is transferred through the walls. This, in turn, has the effect of improving the thermal comfort of the buildings. In addition, the use of these PPW bricks might lead to a reduction in the amount of carbon dioxide produced by operations (CO_2_).

**Figure 15:**
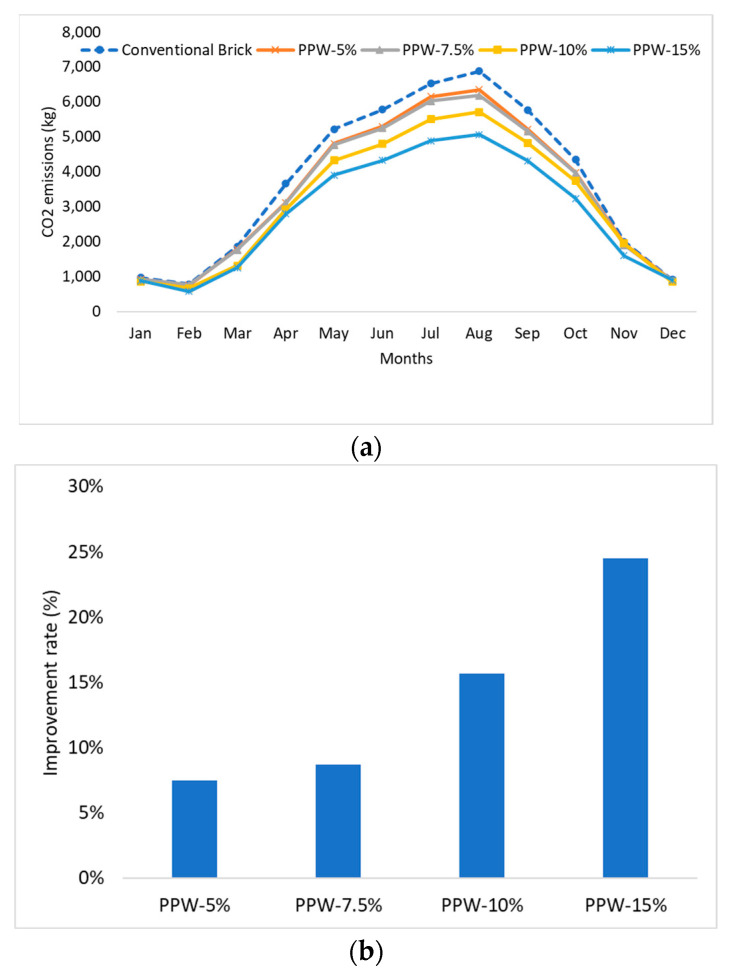
The simulation results for the proposed fabricated clay brick incorporating pomegranate peel waste in terms of; (**a**) monthly CO_2_ emissions, (**b**) the improvement rate.

Figure 15a shows the significant quantities of CO_2_ emissions that are created for the purpose of providing cooling. During the summer, there seems to be a discernible shift in the monthly emissions of CO_2_ on average. One possible explanation for this is the significant amount of energy required to maintain a suitable temperature within the building throughout that period. However, it was shown that making use of PPW-15% could bring in a 24.53% reduction in yearly CO_2_ emissions. In contrast, the PPW-5%, PPW-7.5%, and PPW-10% samples each have the potential to cut yearly CO_2_ emissions by 7.50–8.68% and 15.65%, respectively, as shown in Figure 15b.

The most successful proposed produced brick sample (PPW-15%) was evaluated for thermal and energy efficiency in the city of Jazan, which is located in the Kingdom of Saudi Arabia (Koppen: BSh), which has a hot desert climate. A conventional brick was used as a point of comparison for the sample brick. It is thought that the same model used in Aswan was used in Jazan. PPW-15% saves 31.16% energy. The amount of energy saved is similar to Aswan’s improvement rate. PPW-15% has the potential to reduce CO_2_ emissions by 23.14% in Jazan. Clay bricks created from waste pomegranate peels have the potential to increase thermal performance, energy efficiency, and carbon dioxide emissions in environments that are very hot and dry.

### 3.5. Economic Benefits of Fabricated Brick Samples

The globe is presently shifting toward energy conservation as a result of high expenses. To reduce the amount of heat gained, it was normal practice to insulate the ceilings. However, despite the significant amount of exterior surface area that the walls had, they were often ignored. The purpose of this section is to investigate whether it would be possible to construct conventional bricks using pomegranate waste instead of clay, which would result in significant cost savings.

The results of the study showed that PPW-15% can save energy use by up to 33.13%, which served as the basis for our assessment of the amount of money saved in order to establish whether or not it would be viable to produce pomegranate waste bricks.

Egyptian building owners are always looking for methods to cut their buildings’ operating expenses. Nonetheless, there is always a propensity to reduce the amount of time spent cooling the home with an air conditioner. They also seldom adopt a strategy of depending on passive climate solutions, since the expense of such solutions is frequently an impediment to their implementation. This study looks at the potential financial benefits of using clay bricks made from pomegranate peel waste in low-income housing in Aswan, Egypt.

Even if it is shown that the suggested manufactured brick samples are better suited for energy savings. The study needs to look at the potential and financial gain of these things. As a result, a cost study was conducted, during which the extra investment for each of the suggested brick samples was determined and the total annual energy cost savings were evaluated (in Egyptian Pounds, or EGP).

Regarding this topic, the study compared the prices of a conventional brick wall to those of four brick samples made at a manufacturing facility. A total of 5%, 7.5%, 10%, and 15% of the pomegranate peel wastes were identified in the four samples, respectively. The outlay of finances for the firing mechanism was considered. It was believed that the primary components of building both the conventional brick wall and the suggested brick samples would cost the same. The extra investment and the SPP (in years) were determined to equal [49]:

$$\mathrm{SPP}=\frac{\mathrm{Additional}\;\mathrm{Investment}}{\mathrm{Annual}\;\mathrm{Saving}}$$

The local market provided the production costs (EGP/m^2^) for both the conventional brick wall and the manufactured brick samples. The most up-to-date cost of the components is summarized in Table 5.

Table 6 displays the sums of all the samples’ annual energy costs, total construction costs, annual savings, and SPP. If building owners are looking to save on energy costs, PPW-15% is the best choice. The SPP is 2.26 years, which is the shortest of all the choices. Thus, due to its efficiency and low SPP value, a PPW-15% is the best brick sample among the other proposed manufactured brick samples.

## 4. Conclusions

The primary objective of this study is to analyze the effects of incorporating pomegranate peel waste into clay on the thermal and energy performance of the fabricated bricks. The thermal parameters of significance in this study are specific heat, thermal conductivity, and density. Extensive testing has also been performed on the mechanical and physical characteristics. The study concluded that:

- Adding pomegranate peel waste to brick samples makes them more thermally insulating because it helps form pores, and overall porosity is linked to thermal conductivity.
- The thermal performance of insulated bricks made from the brick sample PPW-15%, which includes 15% of pomegranate peel waste, is the best of all produced brick samples. Its thermal parameters include a density of 1348.25 kg^3^, a specific heat of 1296 J/kg^−1^ K^−1^, and a thermal conductivity of 0.25 Wm^−1^ K^−1^.
- PPW-15% bricks are 33.13% more efficient at preserving cooling energy and produce 24.53% less carbon dioxide emissions yearly compared to conventional bricks. At 1.88 years, the PPW-15% brick sample had the shortest SPP (simple payback period), making it the most cost-effective option.

Lastly, research into how to reuse agricultural waste could lead to the creation of lightweight bricks, which would help reduce CO_2_ emissions by making it easier to cool buildings in hot climates with less energy.

As a result, this study offers an abundance of suggestions that may enhance thermal, energy, and total energy expenses. Instead of conventional bricks, for instance, this research introduces a novel environmental brick sample composed of 15% pomegranate peel waste (PPW-15%). In addition, the national authorities were guided by this research to improve national energy codes and insulation criteria for building envelopes. Furthermore, it draws community attention to the need to meet energy-code regulations during building construction. Finally, this study sheds light on how pomegranate peel waste can be incorporated into fired brick used in the construction of buildings in hot and arid regions, as well as how the findings can be generalized to other places with similar climates.

## Figures and Tables

**Table 1:** Brick samples depending on pomegranate peel waste percentage and firing degree.

Samples	The Percentage of Pomegranate %	Firing Temperature °C
PPW-5%	5	900
PPW-7.5%	7.5	
PPW-10%	10	
PPW-15%	15	

**Table 2:** Characteristics of PPW.

Oxide Composition	Pomegranate Peels Waste wt.%	Clay wt.%
CaO	10.48	0.50
SiO_2_	0.38	48.93
Al_2_O_3_	0.1	32.90
Fe_2_O_3_	0.91	1.19
SO_3_	0.4	0.29
Na_2_O	<0.01	0.09
P_2_O_3_	3.33	-
K_2_O	11.68	0.014
MgO	4.01	0.09
MnO	0.01	-
TiO_2_	0.1	5.92
CL	3.19	0.01
ZrO_2_	-	0.46
Cr_2_O_3_	-	0.14
LOI	65.35	9.2

**Table 3:** The study model input data.

Item	Specification
Building type	Multi-story residential building
Location	New Aswan city—Hot desert region
Building floor area (m^2^)	357
Flat floor area (m^2^)	86
No of Floor	Ground floor and five floors
Floor height (m)	3
Occupancy (person per flat)	5
Window glazing	3 mm single clear glazing
Window-to-wall ratio	10%
Lighting (Lux)	400
HVAC	4 split air conditioning for each flat
Cooling setpoint (°C)	25
Heating setpoint (°C)	18

**Table 4:** The thermal conductivity for each investigated sample.

Sample	Thermal Conductivity (W/m·K)	Density Kg/m^3^	Specific Heat J/kg^−1^ k^−1^
Conventional brick	0.72	1870	800
PPW-5%	0.41	1713.4	1175
PPW-7.5%	0.37	1607	1255
PPW-10%	0.29	1562.8	1288
PPW-15%	0.25	1348.2	1346

**Table 5:** The cost of materials for each unit.

Materials	Material Unit Cost EGP/m^2^
clay-kaolin	50
pomegranate peel wastes	10
Cement mortar	33

**Table 6:** The simple payback period for each option.

	Wall Cost	Additional Investment	Energy Cost	Annual Saving	SPP
	(EGP)	(EGP)	(EGP/year)	(EGP/year)	(year)
Conventional brick	186,750	0	8058.54	0	
PPW-5%	203,625	16,875	6487.47	1571.06	10.74
PPW-7.5%	200,250	13,500	5964.66	2093.87	6.45
PPW-10%	198,000	11,250	5420.65	2637.88	4.26
PPW-15%	192,375	5625	5071.55	2986.98	1.88

## Data Availability

Not applicable.
